# The 2010 Rosetta Developers Meeting: Macromolecular Prediction and Design Meets Reproducible Publishing

**DOI:** 10.1371/journal.pone.0022431

**Published:** 2011-08-31

**Authors:** P. Douglas Renfrew, Gabrielle Campbell, Charlie E. M. Strauss, Richard Bonneau

**Affiliations:** 1 Los Alamos National Labs, Bioscience Division, Los Alamos, New Mexico, United States of America; 2 Computer Science Department, Courant Institute for Mathematical Sciences, New York University, New York, New York, United States of America; 3 Department of Biology, Center for Genomics and Systems Biology, New York University, New York, New York, United States of America; 4 Computational Biology Program, New York University, New York, New York, United States of America; 5 Business Strategy and Development, Association of American Medical Colleges, Washington, D.C., United States of America; 6 The Rosetta Commons, url: http://www.rosettacommons.org/; University of South Florida College of Medicine, United States of America

The Rosetta framework for macromolecular modeling, prediction and design is a widely used code (over 7,000 registered groups) with a large, dynamic developers community (over 200 members). The Rosetta community, including Rosetta developers and users from industry and academia, meets once a year to discuss the science being done to improve Rosetta, new applications of the Rosetta code to biological and chemical problems, and the code development itself. This special collection (RosettaCon 2010) presents a selection of developments in the Rosetta community drawn from RosettaCon 2010. However, this collection is more than a proceedings: this special collection will focus on the publication of *both* the science and the reproducible computational workflow associated with each invited paper's protocol. Each paper in this collection is accompanied by an annotated archive containing the code, scripts and data-sets needed to carry out the computational protocol described in the paper. Example use-cases that directly correspond to each paper are also provided. We call these workflow archives “protocol captures”. By publishing complete protocols and workflows we hope to both publish state-of-the art computational structural biology methods *and* provide an example of how a federated computational collaborative can reproducibly publish and disseminate science based on complex multi-layered protocols.

## Introduction

The complexity and scale of computational biology protocols have tracked the exponential growth of measurements in systems-biology and high-throughput structure determination. Reproduction of the computational protocols underlying important works can be prohibitively difficult outside the constructed computational environment of the original authors. This impedes transmission, pedagogy, and validation. Typical macromolecular modeling protocols involve multiple levels of prediction and design: for example the design of a protein complex that binds a specific DNA sequence will typically iterate amongst algorithms specialized for sequence design, docking, and structure prediction. Many algorithms are stochastic so the results of many Monte-Carlo simulations must be analyzed for ensemble properties. Searches of large sequence databases for homologous proteins [15] may need to be pre-cached or results from multiple secondary structure prediction methods may need to be merged [16]. External codes each require their own installation and databases. Distributing a monolithic code to perform such multi-layered tasks is rarely feasible and might not even allow reproduction if required files or external tools are missing, or complex post-processing steps are required to determine the best designs or models.

In this collection we focus on computational structural biology protocols that use Rosetta [1], [2], [3], [4], [5], [6], [7], [8], [9], [10] [26]. Rosetta was originally developed for *de novo* fold prediction [1], [2], [3], [4], [5] but has been expanded to include methods for design, docking, experimental determination of structure from partial datasets, protein-protein interaction design and prediction, enzyme design, RNA structure prediction and protein-DNA interaction prediction and design [6], [7], [8], [9], [10]. The code is developed by the RosettaCommons. This working collaborative is composed of over 15 academic groups and thus the code is being applied to a very wide diversity of problems. Recent examples of Rosetta's application to challenging problems include: enzyme design, design of novel nucleases, design of new protein topologies, proteome wide de novo structure prediction, prediction and engineering of protein-protein and protein-DNA and protein-surface interfaces, and others. Since these works describe new cutting-edge research, and are not focused solely on the algorithms or workflows employed, the published results typically contain method descriptions with inconsistently stated protocols and dependencies.

RosettaCon 2010 featured three main types of contributions: 1) new features that enabled new applications (vaccine design, enzyme design [11]), 2) new code developments [12], [13] that improve accessibility to the code and support the large development team including a refactoring of the code (Rosetta 3.0) and bindings to popular scripting languages (PyRosetta), and 3) new core scientific developments (multi-state protein design, modeling and design of symmetric protein complexes). This collection aims to make several of these latest Rosetta macromolecular modeling protocols accessible to all. Articles describing 16 of the most important contributions to the conference ranging from new applications, core science, and even reflections on Rosetta's current weaknesses are included in this collection. Frequently an overall process in Rosetta is built from multiple invocations of the Rosetta software with differing command line arguments or inputs, as well as auxiliary tools, specific data-bases, large input data files, and complex post processing steps. Our goal in this special issue is to attempt the capture of all these protocols in a sufficiently complete and formal way that enables outside groups to carry out the complete workflow described in the paper.

It should be noted that this special issue is itself a social experiment (encouraged by the far-looking editors at PLOS) in which we grapple with how best to capture a dynamically evolving set of processes in a way that does not overly burden the authors, works across a distributed community without a central authority for methods capture, is timely, and is sufficiently self-consistent that readers will invest their time in the results. Simply put, if we make the process of capturing protocols too formal and brittle authors and readers will not participate. Not every protocol can be captured as a method from first principles. Not every data set can be encapsulated or kept current automatically. Thus we try to divide processes into incremental sets with defined inputs and outputs. We think that this may advance the baseline in publishing protocols in a robustly reproducible manner for our community and, as we learn from this experience, mature over subsequent efforts by our community.

Each of the articles in this collection are accompanied by an archive containing links to the exact version of the code used in the paper, all input data, links to external tools, and an example script to illustrate the use of the code to carry out the protocol described in the paper. Each paper has this archive (called a protocol capture) as well as a detailed procedural section in the methods section of each paper to describe the proper use of the code with reference to the archived code, scripts, tools and data. Our efforts in this collection are a start on the road to reproducible publication of complex computational analysis. Below we briefly review the history and working structure of the RosettaCommons (the developers body that makes all of this possible), the content of the special issue, and the structure of the Protocol Capture archives that will accompany each article.

## The Rosetta Commons

The RosettaCommons is a multi-group community that develops and maintains Rosetta and also meets regularly to define and coordinate research directions for the Rosetta community (http://www.rosettacommons.org/). The RosettaCommons is a non-profit entity that uses money from licensing the code to industry to support development of the code and dissemination of the code to groups outside the “commons”. Members of RosettaCommons share code pre-publication via a code repository shared by 17 research groups (200 developers), share a large system that validates the large code base with hundreds of scientific benchmarks and unit tests, and have used the RosettaCommons to organize several developer meeting, PI meetings and collaborative works to define the research directions of the group and the labs within the group. The RosettaCommons has allowed the group to re-factor the code completely multiple times without breaking up the constructive collaboration from which we all benefit from.

We describe the RosettaCommons model, not to imply that this is the optimal solution, but to simply describe a solution that has worked for a diverse and dynamic community for over ten years. The Rosetta group, with its needs for real-time sharing and co-development, presented unique challenges to anyone attempting to design an intellectual property structure for this collaborative. These challenges were not met by the standard models in place at the time the collaborative was instantiated. It was clear from early on that the group developing the software was very dynamic and committed to the development project. The multi-institutional nature of the collaborative development, paired with a focus on long-term sustainability, pushed the boundaries of standard licensing programs.

The first two licensing options considered were: release of the code-base under an open source license or a commercially oriented licensing program that allowed non-exclusive licensing. The community had a strong interest in accelerating adoption of the software across the academic and commercial community, but had concern about the complexity of the code and hoped to lead the direction of development. The biggest hurdle to clear from a dissemination standpoint was consolidating all of the rights from developers, and working with the different universities' copyright and patent policies. Moving into an open-source licensing framework would have enabled adoption by academic users, but, depending on the license used, may have made any control of the software development rather challenging. The complexity of the code also raised concerns about releasing the source code for modification and the potential negative implications on use and performance. Lastly, the group felt (at the inception of the collaborative in 2001) that it would be difficult to fund code maintenance activities, and that without the ability to generate revenue for the licensing and supporting of Rosetta modules the effort would flounder.

In conjunction with multiple institutions, the University of Washington led the creation of a formal community of developers that allowed open-sharing and modifications of code among the Rosetta Commons participants. Significantly while the barrier for entry to the Commons was kept low, license agreements to those outside the community maintained control over how the software was used and/or modified and restricted any further dissemination. To foster this community foundation, all developers waived personal royalties and instead directed their royalty shares to the Rosetta Commons, a central management point for the community devoted to the development and dissemination of the code. These funds were used to support a project manager, shared hardware, and the annual Rosetta Commons meeting attended by Rosetta Commons contributors. Individuals who contributed to the code base were required to sign an agreement acknowledging ground rules and copyright assignment (by their respective institutions). Institutions with developers contributing to the code base agreed, on joining the commons, to waive their right to assert intellectual property rights against any Rosetta developers or licensees. Those institutions maintained ownership of the copyright and patents and granted permission to the University of Washington for licensing the software modules to additional academic and commercial users. These agreements provided a foundation for the intellectual property and licensing framework.

With this framework in place the academic and commercial licensing program quickly developed as the code matured and the community diversified (academics were also immediately provided free access to the source code). The commercial licensing program generated revenue that contributed support to a project manager and the annual Rosetta developers meeting in Leavenworth, Washington, including travel costs for all attendees. Feedback from commercial and academic users provided the developers with a test community for development. With the academic users able to use the source code (for free) but not disseminate it, the Rosetta Commons maintained control over the code base while enabling adoption across the academic community. The high expectations of the Rosetta Commons participants from each other, the commitment of the developers to the Rosetta Commons community, and the willingness of the universities to enter an unusual multi-university software development collaborative (while waiving royalties from commercial licensing) enabled the success of this community framework.

## RosettaCon 2010, code, science, and applications

This special issue focuses on three types of papers described briefly below. In each area we highlight one of the papers to demonstrate how the protocol capture (the workflow) will aid other groups trying to carry out similar analysis. In each case we highlight the aspects of the workflow beyond just installing Rosetta and the command line arguments.

### New Rosetta applications

Several articles describe specific applications in biology or chemistry, including *de novo* enzyme design, modeling classes of protein loops, design of temperature sensitive mutations, and design of peptides to inhibit large surface area protein interactions. The article describing the design of temperature sensitive mutations is a prime example of the need for the publishing of complete workflows: models of target proteins are made, models for all possible mutations are relaxed (to accommodate mutations) and scored, and lastly a machine learning procedure is used to select mutations likely to confer a temperature sensitive behavior to the protein. The protocol capture associated with this paper contains all auxiliary code (including scripts to make initial models and machine learning code and packages).

### Rosetta basic science

A main focus of the Rosetta developers meeting is the development of new functionalities and the improvement of existing capabilities. The 2010 meeting was no exception, and several of the contributions in this collection are Rosetta basic science papers of this type. Examples include: incorporation of non-canonical amino acids in Rosetta design, multi-state design, new Rosetta kinematics, new protein docking protocols, and anchored design. A particularly interesting paper in this vein is the paper by Das (“Four small puzzles that Rosetta can't solve”) provides four very clear examples of where Rosetta structure prediction needs to be improved. Each example has the full protocol that lead to the incorrect prediction fully described as well as the correct (“native”) structure; thus these protocols are key elements in defining and judging future improvement in Rosetta and other codes.

### Rosetta code development

Multiple articles in this collection describe new code refactoring, extensions or improvements to the implementation of Rosetta. Several articles discuss the creation of multi-purpose high level interfaces to the components of Rosetta. Examples include an XML scripting interface for Rosetta, an interactive python interface to the Rosetta code, and an object oriented API for generating Rosetta fragments. Rosetta uses, as part of several protocols, fragments of other known structures. These fragments are particularly useful for modeling long loops and protein structure prediction. Gront et al. describe a new code for predicting fragments. Having a published workflow for this step, building fragments, is important, as it is core to several of the published protocols and is nontrivial (involving a specialized database [14], auxiliary tools such as PSI-BLAST [15] and PSIPRED [16] and has several configurable options).

### Reproducible methods via capture of multi-level computational macromolecular prediction and design protocols

This special collection provides the larger community direct access to the exact protocols used in each of the papers in this collection. The construction is intended to 1) allow other community members and Rosetta users to exactly reproduce the work; 2) allow competing groups to validate and improve upon on the work; 3) make it rapidly accessible to new users with similar biological applications. The area of enabling reproducible publishing of science is not new and much recent work has focused on several ideas for how to facilitate reproducible scientific publishing. Those ideas can be divided loosely into two main areas of work: 1) reproducible publication of data and results linked to publications [17], [18], [19] and 2) reproducible publication of workflows and analysis performed as part of a publication (e.g., Vistrails, myExperiment, Open Lab Book) [20], [21], [22], [23], [24], [25]. This issue focuses on the second set of issues, the publication of all aspects of complex structural bioinformatics protocols.

There are several well-developed sets of ideas in this domain and we focus on one solution out a great many possible solutions. As suggested in the SIGMOD 2011 Repeatability Experiment (http://www.sigmod2011.org/calls_papers_sigmod_research_repeatability.shtml) best practice solutions to the problem of reproducibly publishing workflows and complex computational protocols follow a spectrum that, very roughly, progresses from simple retrospective solutions that can be implemented after a analysis has been performed (e.g., a comprehensive archive containing a script that executes the workflow and an archive of all inputs and example outputs for error checking), to systems that automatically log and manage computational workflows as they are created and provide automated tools for sharing, comparing, and searching workflows. These solutions provide systems for creating workflows that allow developers to add new procedures and (in some cases) construct and test new workflows via a graphical user interface [24]. Another possible solution is to perform all analysis for a paper or other work on a virtual machine (e.g., virtual box, http://www.virtualbox.org/) and then distribute the virtual machine (essentially ensuring that all OS, file system, inputs, and other dependencies are exactly identical between the described analysis and the one that is distributed). This approach has the advantage of being the *exact* analysis performed, but the disadvantage of perpetuating hidden or accidental dependencies.

In this case we chose the retrospective solution and each paper will be accompanied by a protocol capture. Each protocol capture conforms to a template distributed to all authors that contains each of the following template files or directories:

## INSTRUCTIONS

This is a text file with simple instructions for populating the temple and testing the execution of each script and Rosetta command line. Instructions include requirements for auxiliary code and Rosetta version identifiers.

### inputs/

A directory in the archive containing all data needed for the example run. It is important to note that each archive will not be limited by the disk-space limits usually placed on supplemental material (e.g., if the author used a search of all structures in the PDB then this database will be included in the input/section of the protocol capture).

### outputs/

All output of scripts and Rosetta as structured in the analysis described in the paper. It is expected that this be the result of the example script and command lines (x Rosetta version) given in the scripts/directory of the archive.

## README

Authors provide a README file that describe the sequential use of the protocol scripts, auxiliary data-files and dependencies. References and instructions for building the code in the repository are also provided.

## run_example (multiple)

Although the scripts/directory provides the main capture of the protocol and is the main mechanism allowing people to reproduce the work, we also require authors to provide Rosetta command lines as a means of helping readers to isolate Rosetta specific aspects of the protocol (as these will be a major focus of each paper).

## scripts/

This directory will contain scripts (nested in many cases) that execute the analysis described in the paper. This section is typically the most useful section of the protocol capture. All protocols will be verified as part of the review process to ensure that the scripts provided run properly on computers other than the authors.

## UPLOAD

This text file provides instructions for uploading the archive to the Rosetta Commons source code repository (which will serve the protocol captures in parallel with the journal website).

For an example protocol capture see the supplemental sections of the articles in this collection. For the simple template we distributed to all authors see the supplement to this article. Several of the papers in this collection describe multiple, nested or forked workflows and in these cases multiple validated protocol captures are provided.

Methods sections for each paper should also include a section entitled “Detailed Workflow” that described the protocol capture and carefully walks the reader through using the code via example command lines and references to the protocol capture that are sequential and complete. The regular methods section should be extensive and refer to the code and the workflow more than is usual for a research article. Think “Joy of Cooking”.

## Conclusion

As organizers of this collection we want to thank the Rosetta Community for embracing our attempt to enhance the reproducibility of published applied computational research. The articles presented here could have been published elsewhere on their own scientific merits at the cost of streamlined, less accessible, methods. By agreeing to adopt a consistent set of publication standards for capturing their protocols these authors have created something that we hope will be highly accessible to readers interested in actually using the methods for themselves. Although this special issue falls short of the ultimate reproducible publication solutions that are on the horizon we hope that this effort will help establish the value of the practical, user-centric, capture of complex state of the art macromolecular modeling and design computational protocols. It is inevitable that many successful federated enterprises will require retrospective capture during their periods of most active research. By linking a retrospective, but reproducible executable, archive to each paper, and by annotating each protocol we hope to ensure that all papers in this collection are completely reproducible by outside groups. We have tried to divide long pipelines into stages, each with defined inputs and outputs, as future applications are likely to require just certain components of any existing pipeline. We hope that this and similar models can link the reward of publishing with the reproducible dissemination of code and workflows. In many ways our solution is a social construct built in part on a solid base of constructive collaborative spirit (i.e. the RosettaCommons). It is difficult to say what is next for the Rosetta developers community. It less difficult to predict that as we continue to grow and face new implementation, funding, dissemination and (most importantly) scientific challenges, we will face them together and we will continue to strive to make the work more reproducibly accessible. For a current list of Rosetta developers and participating labs see: http://www.rosettacommons.org/.
